# Crystal structure of 1-{4-[bis­(4-methyl­phen­yl)amino]­phen­yl}ethene-1,2,2-tricarbo­nitrile

**DOI:** 10.1107/S2056989024001804

**Published:** 2024-02-29

**Authors:** Mamoun M. Bader, Phuong-Truc Pham

**Affiliations:** a Alfasial University, Riyadh, Saudi Arabia; b Penn State Scranton, Dunmore, PA, USA; Institute of Chemistry, Chinese Academy of Sciences

**Keywords:** crystal structure, donor/acceptor, dyes, tri­phenyl­amine, tri­cyano­vin­yl

## Abstract

The title compound, C_25_H_18_N_4_, crystallizes in the centrosymmetric ortho­rhom­bic space group *Pbca*, with eight mol­ecules in the unit cell. The main feature noticeable in the structure is the impact of the tri­cyano­vinyl (TCV) group in forcing partial planarity of the portion of the mol­ecule carrying the TCV group and directing the mol­ecular packing in the solid state, resulting in the formation of π-stacks of dimers within the unit cell. Short π–π stack closest atom-to-atom distances of 3.444 (15) Å are observed. Such motif patterns are favorable as they are thought to be conducive for better charge transport in organic semiconductors, which results in enhanced device performance. Intra­molecular charge transfer is evident from the shortening in the observed experimental bond lengths. The nitro­gen atoms (of the cyano groups) are involved in extensive short contacts, primarily through C—H⋯NC inter­actions with distances of 2.637 (17) Å.

## Abstract 2

The title compound crystallizes in the centrosymmetric ortho­rhom­bic space group *Pbca*, with 8 mol­ecules in the unit cell. The main feature noticeable in the structure is the impact of the tri­cyano­vinyl (TCV) group in forcing partial planarity of the portion of the mol­ecule carrying the TCV group and directing the mol­ecular packing in the solid state, resulting in the formation of π-stacks of dimers within the unit cell.

## 1. Chemical context

Tri­phenyl­amine and its derivatives have been employed in a wide range of applications in materials chemistry. Some of the most exploited applications of this important building block include: hole-transport materials, organic light-emitting diodes, photoconductors, photodiodes, semiconductors, and solar cell applications. The optical properties of tri­phenyl­amine derivatives have been explored in optical telecommunications, optical data storage, laser frequency conversion, color displays, and non-linear optics including optical power limiters and multiphoton absorption (Khasbaatar *et al.*, 2023 ▸; Kong *et al.*, 2012 ▸; Itoo *et al.*, 2022 ▸; Bian 2023 ▸). In particular, donor/acceptor mol­ecules incorporating this building block have received considerable attention. Synthetically, many creative and inter­esting mol­ecular architectures incorporating tri­phenyl­amines have been reported (El-Nahass *et al.*, 2013 ▸; Ogunyemi *et al.*, 2020 ▸).

Both mol­ecular design and solid-state structures are important in effectively using mol­ecular materials in the above-mentioned applications. Highly conjugated mol­ecules with delocalized electrons synthesized by systematic modifications allow for access to a wide range of structures. However, the way the mol­ecules are arranged in the solid state, either in thin films or in single crystals, dictates the performance of devices built with these mol­ecular materials. Attention to solid-state structures of organic functional materials has steadily gained momentum. Much more work is still needed in this area to help better understand the competing inter- and intra­molecular inter­actions in determining their solid-state structures. This study focuses on one the impact of the presence of the tri­cyano­vinyl group on the solid-state structure of the title compound, which is also compared with those of closely related structures. 
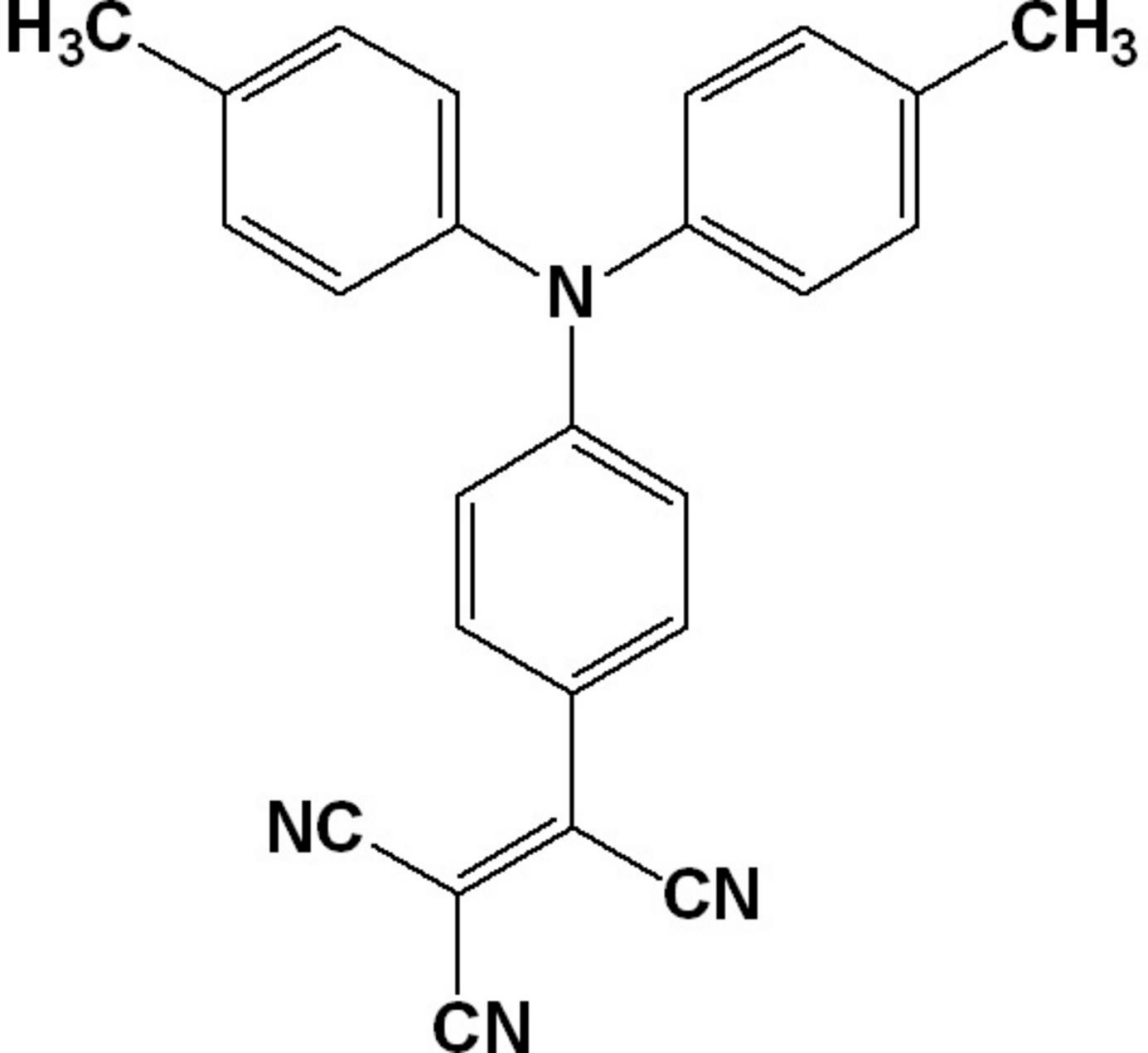


## 2. Structural commentary

The crystal structure of tri­phenyl­amine is known and has been examined several times (Martin *et al.*, 2007 ▸; Sobolev *et al.*, 1985 ▸; Howells *et al.*, 1954 ▸) There are no significant close inter­actions within the unit cell of tri­phenyl­amine except for C—H⋯π with a relatively long distance (2.817Å). We also note that there have been several recent structural reports on tri­phenyl­amine derivatives, with various structural features including multi­cyano­derivatives (Ishi *et al.*, 2019 ▸; Akahane *et al.*, 2018 ▸; Hariharan *et al.*, 2017 ▸; Song *et al.*, 2006 ▸; Tang *et al.*, 2010 ▸).

**Figure 1:**
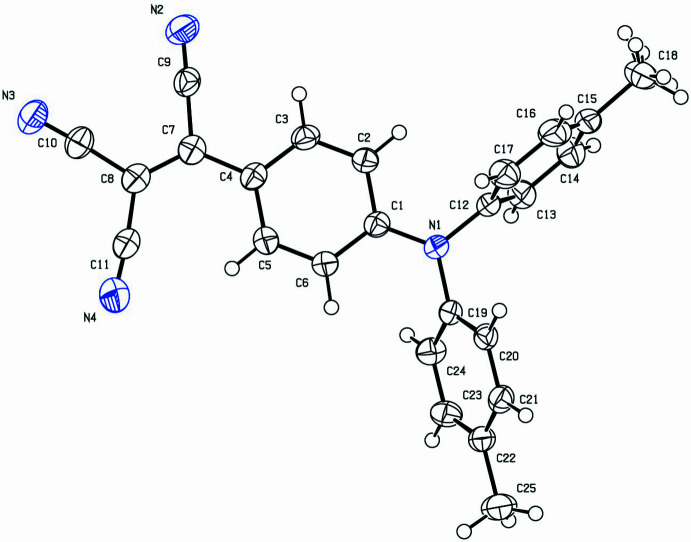
The mol­ecule in the crystal. Ellipsoids represent 50% probability levels.

**Figure 2:**
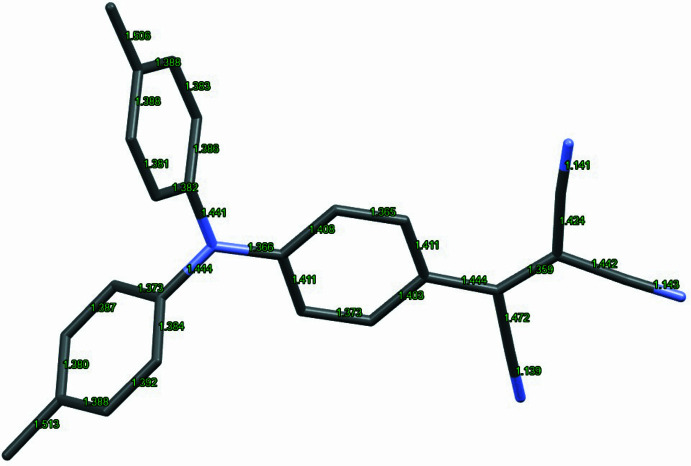
Bond lengths indicating charge-transfer inter­actions in the title compound.

The closest reported structures to the title compound are the corresponding mol­ecule without the methyl groups tri­cyano­vinyl­tri­phenyl­amine, which we will refer to as Ph_3_N-TCV (CYVTPA; Vozzhennikov *et al.*, 1979 ▸; Popova *et al.*, 1976 ▸, 1977 ▸). It is worth mentioning that the title compound forms shiny metallic crystals with large smooth surfaces·We note that, as expected, the title compound adopts a propeller mol­ecular shape and crystallizes in the ortho­rhom­bic space group *Pbca*, similar to Ph_3_N-TCV. (Fig. 1 ▸) The angles around the central nitro­gen atom are all nearly the same, showing similar trends, with the smallest angle between the phenyl groups without the electron-accepting group: 116.71 (14), 120.27 (14), 123.02 (15)° in the title compound Me2-Ph_3_N-TCV and 116, 121, 123° in Ph_3_N-TCV, whereas the C—N bond lengths are clearly significantly shorter for the ring bearing the electron acceptor. Almost identical lengths are observed in this structure and Ph_3_N-TCV: *1.366 (2)*, 1.441 (2), 1.444 (2) Å in the title compound compared with *1.38*, 1.44, 1,44 Å in Ph_3_N-TCV. The shortest lengths (depicted in *italics*) are for the N—C bond on the phenyl ring carrying the TCV groups, suggesting, as expected, intra­molecular charge transfer (Fig. 2 ▸). The angles around the central nitrogen atom indicate planarity and range from to 116.71 (14) to 123.02 (15)°.

## 3. Supra­molecular features

**Figure 3:**
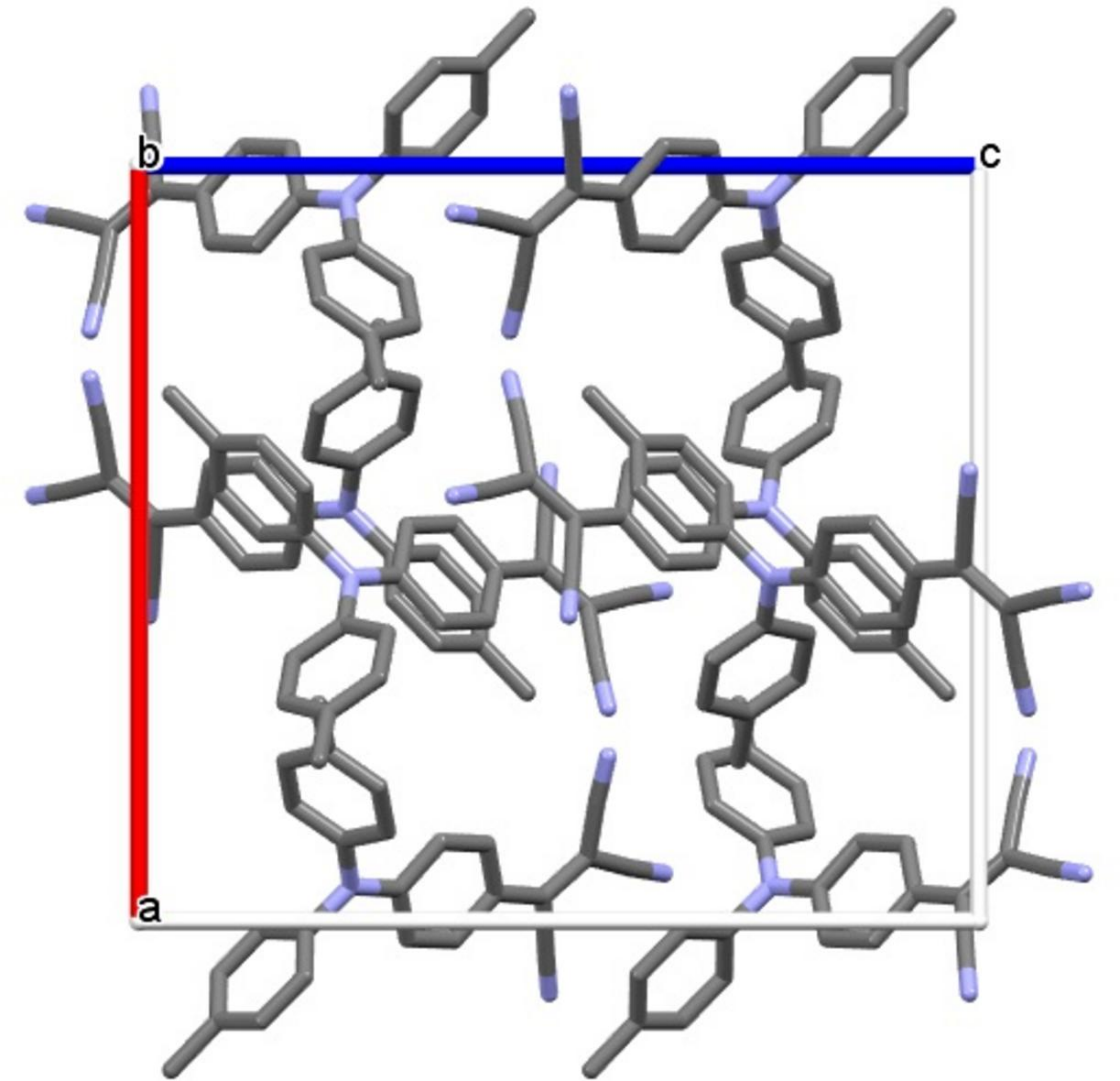
Unit cell of the title compound.

**Figure 4:**
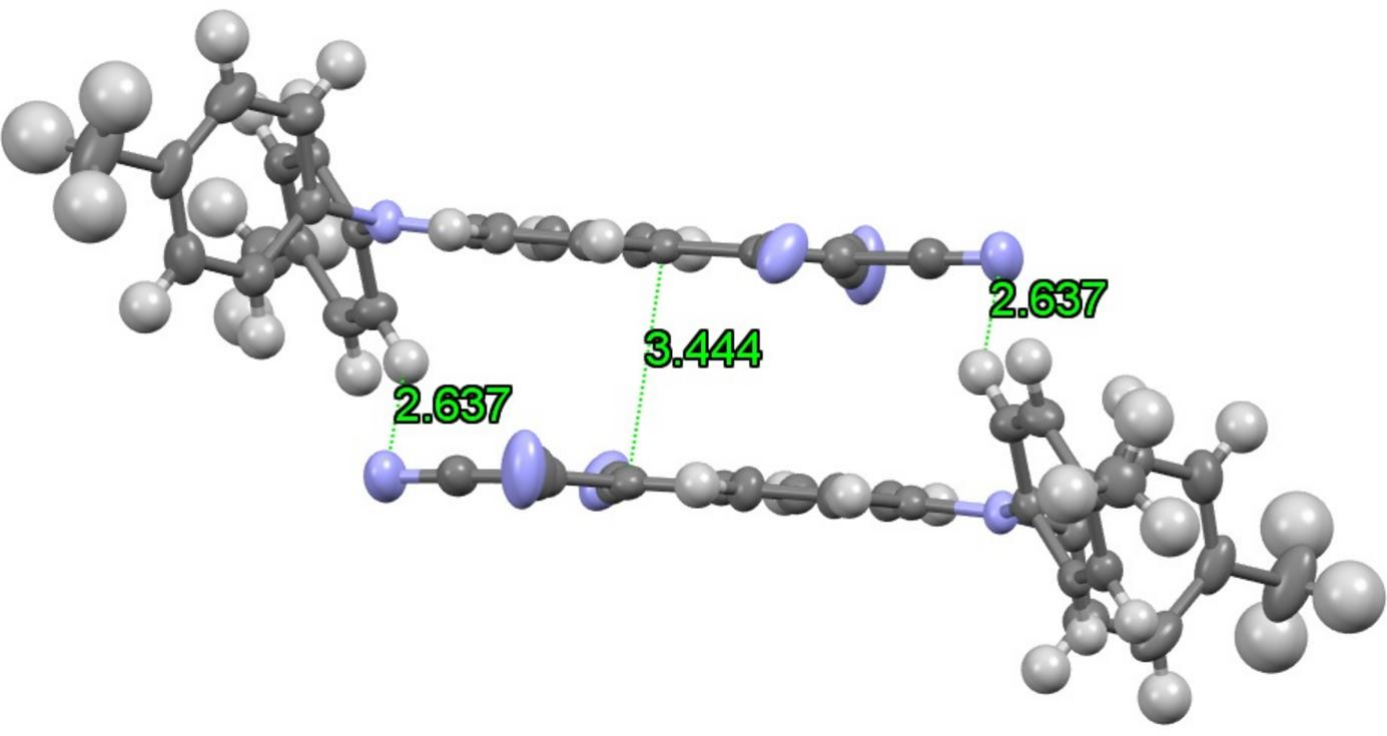
π-Stacking and C—H⋯N inter­actions in the title compound.

In the crystal (Fig. 3 ▸), the mol­ecules form π-stacked dimers involving the acceptor-carrying phenyl rings of two adjacent mol­ecules with a shortest atom-to-atom distance of 3.444 (15) Å, which compares with 3.616 Å in Ph_3_N-TCV. The dimers are further held together by C—H⋯NC inter­actions on both ends (Fig. 4 ▸). With distances of 2.637 (17) Å, the inter­actions in the title compound are slightly weaker than those observed in Ph_3_N-TCV (2.462 Å).

## 4. Database survey

A survey of the Cambridge Structural Database (CSD; Groom *et al.*, 2016 ▸) in February 2024 revealed more than 30 hits each for ‘tri­phenyl­amine’ and ‘tri­cyano­vin­yl’. No hits were found for the title compound. The closely related structure for a similar compound without the methyl groups (Popova *et al.*, 1977 ▸) is compared with the title compound above.

## 5. Synthesis and crystallization

*N*,*N*-*p*-di­tolyl­aniline (Aldrich, 0.5 mmol) was reacted with tetra­cyano­ethyl­ene (TCNE, Aldrich, 0.75 mmol) in DMF (5 mL) in a 25 mL round-bottom flask at room temperature. After 2 h the reaction was worked out either by addition of 6 *M* HCl or extraction by methyl­ene chloride. The product was isolated as a purple solid, m.p. 462–463 K, and crystallized by slow evaporation from aceto­nitrile. ^1^H NMR, ppm: 7.13 (*d*, 6H); 7.15 (*d*, 4H); 7.30 (*d*, 2H); 2.32 (*s*, 6H).

## 6. Refinement

Crystal data, data collection and structure refinement details are summarized in Table 1 ▸. H atoms were positioned geometrically (C—H = 0.95–0.98 Å) and refined as riding with *U*_iso_(H) = 1.2*U*_eq_(C) or 1.5*U*_eq_(C-meth­yl).

## Supplementary Material

Crystal structure: contains datablock(s) I. DOI: 10.1107/S2056989024001804/nx2005sup1.cif

Structure factors: contains datablock(s) I. DOI: 10.1107/S2056989024001804/nx2005Isup2.hkl

Supporting information file. DOI: 10.1107/S2056989024001804/nx2005Isup3.cml

CCDC reference: 2202337

Additional supporting information:  crystallographic information; 3D view; checkCIF report

## Figures and Tables

**Table 1:** Experimental details

Crystal data	
Chemical formula	C_25_H_18_N_4_
*M*_r_	374.43
Crystal system, space group	Orthorhombic, *P* *b* *c* *a*
Temperature (K)	173
*a*, *b*, *c* (Å)	16.8662 (15), 12.8555 (11), 18.7561 (16)
*V* (Å^3^)	4066.8 (6)
*Z*	8
Radiation type	Mo *K*α
μ (mm^−1^)	0.07
Crystal size (mm)	0.35 × 0.32 × 0.03

Data collection	
Diffractometer	Bruker APEXII CCD
Absorption correction	Multi-scan (*SADABS*; Krause *et al.*, 2015 ▸)
*T*_min_, *T*_max_	0.975, 0.998
No. of measured, independent and observed [*I* > 2σ(*I*)] reflections	23265, 4170, 2586
*R*_int_	0.057
(sin θ/λ)_max_ (Å^−1^)	0.626

Refinement	
*R*[*F* ^2^ > 2σ(*F* ^2^)], *wR*(*F* ^2^), *S*	0.048, 0.125, 1.02
No. of reflections	4170
No. of parameters	264
H-atom treatment	H-atom parameters constrained
Δρ_max_, Δρ_min_ (e Å^−3^)	0.39, −0.19

Computer programs: *APEX2* and *SAINT* (Bruker, 2014 ▸), *SHELXS97* (Sheldrick, 2008 ▸), *SHELXL2018/3* (Sheldrick, 2015 ▸) and *SHELXTL* (Sheldrick, 2008 ▸).

## supplementary crystallographic information

### Crystal data

**Table:**

C_25_H_18_N_4_	*D*_x_ = 1.223 Mg m^−^^3^
*M**_r_* = 374.43	Mo *K*α radiation, λ = 0.71073 Å
Orthorhombic, *P**b**c**a*	Cell parameters from 2998 reflections
*a* = 16.8662 (15) Å	θ = 2.2–24.6°
*b* = 12.8555 (11) Å	µ = 0.07 mm^−^^1^
*c* = 18.7561 (16) Å	*T* = 173 K
*V* = 4066.8 (6) Å^3^	Plate, red
*Z* = 8	0.35 × 0.32 × 0.03 mm
*F*(000) = 1568	

### Data collection

**Table:**

Bruker APEXII CCD diffractometer	2586 reflections with *I* > 2σ(*I*)
Radiation source: sealed tube	*R*_int_ = 0.057
φ and ω scans	θ_max_ = 26.4°, θ_min_ = 2.2°
Absorption correction: multi-scan (SADABS; Krause *et al.*, 2015)	*h* = −21→21
*T*_min_ = 0.975, *T*_max_ = 0.998	*k* = −15→16
23265 measured reflections	*l* = −23→13
4170 independent reflections	

### Refinement

**Table:**

Refinement on *F*^2^	0 restraints
Least-squares matrix: full	Hydrogen site location: inferred from neighbouring sites
*R*[*F*^2^ > 2σ(*F*^2^)] = 0.048	H-atom parameters constrained
*wR*(*F*^2^) = 0.125	*w* = 1/[σ^2^(*F*_o_^2^) + (0.047*P*)^2^ + 1.2157*P*] where *P* = (*F*_o_^2^ + 2*F*_c_^2^)/3
*S* = 1.02	(Δ/σ)_max_ < 0.001
4170 reflections	Δρ_max_ = 0.39 e Å^−^^3^
264 parameters	Δρ_min_ = −0.19 e Å^−^^3^

### Special details

**Table:**

Geometry. All esds (except the esd in the dihedral angle between two l.s. planes) are estimated using the full covariance matrix. The cell esds are taken into account individually in the estimation of esds in distances, angles and torsion angles; correlations between esds in cell parameters are only used when they are defined by crystal symmetry. An approximate (isotropic) treatment of cell esds is used for estimating esds involving l.s. planes.

### Fractional atomic coordinates and isotropic or equivalent isotropic displacement parameters (Å^2^)

**Table:**

	*x*	*y*	*z*	*U*_iso_*/*U*_eq_	Occ. (<1)
N1	0.54561 (9)	0.35769 (12)	0.25270 (8)	0.0327 (4)	
N2	0.40435 (13)	0.75779 (16)	0.48843 (10)	0.0608 (6)	
N3	0.56270 (11)	0.81259 (15)	0.62522 (10)	0.0520 (5)	
N4	0.71782 (13)	0.57500 (19)	0.55835 (12)	0.0784 (7)	
C1	0.54228 (11)	0.42447 (14)	0.30928 (10)	0.0309 (4)	
C2	0.47659 (11)	0.49077 (14)	0.31939 (10)	0.0341 (4)	
H2A	0.433200	0.487458	0.287170	0.041*	
C3	0.47442 (12)	0.56000 (15)	0.37507 (10)	0.0360 (5)	
H3A	0.429465	0.603864	0.380322	0.043*	
C4	0.53662 (11)	0.56791 (14)	0.42448 (10)	0.0331 (4)	
C5	0.60170 (11)	0.50065 (15)	0.41415 (10)	0.0380 (5)	
H5A	0.644920	0.503697	0.446554	0.046*	
C6	0.60455 (11)	0.43136 (15)	0.35909 (10)	0.0367 (5)	
H6A	0.649299	0.387029	0.354280	0.044*	
C7	0.53210 (12)	0.64360 (15)	0.48125 (10)	0.0359 (5)	
C8	0.58477 (12)	0.66465 (15)	0.53418 (11)	0.0402 (5)	
C9	0.46017 (13)	0.70824 (16)	0.48442 (10)	0.0401 (5)	
C10	0.57010 (12)	0.74718 (17)	0.58452 (11)	0.0418 (5)	
C11	0.65825 (14)	0.61249 (18)	0.54528 (12)	0.0487 (6)	
C12	0.48557 (11)	0.36131 (14)	0.19788 (9)	0.0319 (4)	
C13	0.42598 (12)	0.28855 (16)	0.19709 (11)	0.0408 (5)	
H13A	0.424981	0.234803	0.231836	0.049*	
C14	0.36727 (13)	0.29368 (18)	0.14546 (12)	0.0491 (6)	
H14A	0.326013	0.243369	0.145631	0.059*	
C15	0.36736 (13)	0.37017 (19)	0.09383 (11)	0.0479 (6)	
C16	0.42856 (14)	0.44230 (18)	0.09488 (11)	0.0524 (6)	
H16A	0.430298	0.495020	0.059426	0.063*	
C17	0.48744 (13)	0.43899 (16)	0.14681 (11)	0.0438 (5)	
H17A	0.528520	0.489550	0.147200	0.053*	
C18	0.30260 (15)	0.3765 (2)	0.03817 (13)	0.0773 (9)	
H18A	0.279705	0.307222	0.030804	0.116*	0.5
H18B	0.324991	0.401877	−0.006783	0.116*	0.5
H18C	0.261172	0.424333	0.054453	0.116*	0.5
H18D	0.297540	0.448399	0.021512	0.116*	0.5
H18E	0.252254	0.353745	0.059098	0.116*	0.5
H18F	0.316073	0.331288	−0.002137	0.116*	0.5
C19	0.60719 (10)	0.28093 (14)	0.24428 (10)	0.0297 (4)	
C20	0.65333 (10)	0.28135 (14)	0.18337 (10)	0.0313 (4)	
H20A	0.645477	0.333290	0.148039	0.038*	
C21	0.71086 (11)	0.20612 (15)	0.17394 (10)	0.0358 (5)	
H21A	0.741966	0.206892	0.131715	0.043*	
C22	0.72438 (11)	0.12945 (14)	0.22459 (10)	0.0344 (5)	
C23	0.67765 (11)	0.13075 (15)	0.28548 (11)	0.0391 (5)	
H23A	0.685992	0.079460	0.321166	0.047*	
C24	0.61923 (11)	0.20484 (15)	0.29552 (11)	0.0376 (5)	
H24A	0.587496	0.203584	0.337350	0.045*	
C25	0.78680 (13)	0.04717 (17)	0.21395 (13)	0.0520 (6)	
H25A	0.799588	0.041696	0.163120	0.078*	
H25B	0.766801	−0.019848	0.231175	0.078*	
H25C	0.834624	0.066109	0.240628	0.078*	

### Atomic displacement parameters (Å^2^)

**Table:**

	*U*^11^	*U*^22^	*U*^33^	*U*^12^	*U*^13^	*U*^23^
N1	0.0356 (9)	0.0306 (9)	0.0321 (9)	0.0070 (7)	−0.0031 (7)	−0.0046 (7)
N2	0.0746 (14)	0.0627 (13)	0.0451 (12)	0.0302 (12)	−0.0004 (10)	−0.0061 (10)
N3	0.0542 (12)	0.0501 (12)	0.0516 (12)	−0.0003 (9)	0.0070 (9)	−0.0149 (10)
N4	0.0614 (14)	0.0978 (18)	0.0760 (16)	0.0188 (13)	−0.0223 (12)	−0.0394 (14)
C1	0.0368 (10)	0.0253 (10)	0.0306 (10)	0.0008 (8)	0.0011 (8)	0.0027 (8)
C2	0.0379 (10)	0.0318 (11)	0.0327 (10)	0.0059 (9)	−0.0030 (9)	0.0007 (9)
C3	0.0414 (11)	0.0307 (11)	0.0358 (11)	0.0083 (9)	0.0019 (9)	0.0019 (9)
C4	0.0428 (11)	0.0269 (10)	0.0297 (10)	0.0013 (9)	0.0041 (8)	0.0020 (8)
C5	0.0387 (11)	0.0431 (12)	0.0323 (11)	0.0028 (9)	−0.0047 (9)	−0.0040 (9)
C6	0.0359 (10)	0.0396 (12)	0.0347 (11)	0.0085 (9)	−0.0029 (9)	−0.0041 (9)
C7	0.0451 (11)	0.0314 (11)	0.0312 (11)	−0.0021 (9)	0.0034 (9)	0.0050 (8)
C8	0.0450 (12)	0.0358 (12)	0.0396 (12)	0.0010 (10)	0.0041 (9)	−0.0019 (9)
C9	0.0551 (13)	0.0352 (12)	0.0301 (11)	0.0061 (11)	−0.0014 (10)	−0.0005 (9)
C10	0.0459 (12)	0.0398 (12)	0.0398 (12)	−0.0044 (10)	0.0080 (10)	−0.0038 (10)
C11	0.0493 (14)	0.0481 (14)	0.0486 (14)	0.0057 (11)	−0.0028 (11)	−0.0173 (11)
C12	0.0361 (10)	0.0312 (11)	0.0285 (10)	0.0083 (9)	−0.0021 (8)	−0.0038 (8)
C13	0.0434 (11)	0.0406 (12)	0.0385 (12)	0.0026 (10)	−0.0042 (9)	0.0004 (9)
C14	0.0419 (12)	0.0553 (15)	0.0502 (14)	0.0016 (11)	−0.0068 (10)	−0.0124 (11)
C15	0.0430 (12)	0.0658 (16)	0.0348 (12)	0.0242 (12)	−0.0065 (10)	−0.0147 (11)
C16	0.0652 (15)	0.0573 (15)	0.0348 (12)	0.0259 (13)	0.0006 (11)	0.0071 (11)
C17	0.0490 (12)	0.0405 (12)	0.0419 (12)	0.0041 (10)	−0.0013 (10)	0.0059 (10)
C18	0.0597 (16)	0.121 (2)	0.0513 (16)	0.0463 (16)	−0.0174 (12)	−0.0201 (16)
C19	0.0308 (9)	0.0269 (10)	0.0314 (10)	0.0009 (8)	−0.0033 (8)	−0.0040 (8)
C20	0.0354 (10)	0.0268 (10)	0.0315 (10)	−0.0008 (8)	−0.0038 (8)	−0.0019 (8)
C21	0.0353 (10)	0.0377 (12)	0.0343 (11)	−0.0004 (9)	0.0028 (9)	−0.0054 (9)
C22	0.0298 (10)	0.0300 (11)	0.0434 (12)	0.0001 (8)	−0.0023 (9)	−0.0050 (9)
C23	0.0395 (11)	0.0321 (11)	0.0456 (12)	0.0037 (9)	−0.0021 (10)	0.0081 (9)
C24	0.0379 (11)	0.0384 (12)	0.0364 (11)	0.0033 (9)	0.0058 (9)	0.0046 (9)
C25	0.0455 (12)	0.0478 (14)	0.0627 (15)	0.0147 (11)	0.0015 (11)	−0.0018 (12)

### Geometric parameters (Å, º)

**Table:**

N1—C1	1.366 (2)	C14—H14A	0.9500
N1—C19	1.441 (2)	C15—C16	1.388 (3)
N1—C12	1.444 (2)	C15—C18	1.513 (3)
N2—C9	1.139 (3)	C16—C17	1.392 (3)
N3—C10	1.143 (2)	C16—H16A	0.9500
N4—C11	1.141 (3)	C17—H17A	0.9500
C1—C6	1.409 (3)	C18—H18A	0.9800
C1—C2	1.411 (2)	C18—H18B	0.9800
C2—C3	1.373 (3)	C18—H18C	0.9800
C2—H2A	0.9500	C18—H18D	0.9800
C3—C4	1.404 (3)	C18—H18E	0.9800
C3—H3A	0.9500	C18—H18F	0.9800
C4—C5	1.411 (3)	C19—C20	1.382 (2)
C4—C7	1.444 (3)	C19—C24	1.386 (3)
C5—C6	1.365 (3)	C20—C21	1.381 (2)
C5—H5A	0.9500	C20—H20A	0.9500
C6—H6A	0.9500	C21—C22	1.388 (3)
C7—C8	1.359 (3)	C21—H21A	0.9500
C7—C9	1.472 (3)	C22—C23	1.388 (3)
C8—C11	1.424 (3)	C22—C25	1.506 (3)
C8—C10	1.442 (3)	C23—C24	1.383 (3)
C12—C13	1.373 (3)	C23—H23A	0.9500
C12—C17	1.384 (3)	C24—H24A	0.9500
C13—C14	1.387 (3)	C25—H25A	0.9800
C13—H13A	0.9500	C25—H25B	0.9800
C14—C15	1.380 (3)	C25—H25C	0.9800
			
C1—N1—C19	123.02 (15)	C16—C17—H17A	120.3
C1—N1—C12	120.27 (14)	C15—C18—H18A	109.5
C19—N1—C12	116.71 (14)	C15—C18—H18B	109.5
N1—C1—C6	121.61 (16)	H18A—C18—H18B	109.5
N1—C1—C2	121.09 (16)	C15—C18—H18C	109.5
C6—C1—C2	117.29 (17)	H18A—C18—H18C	109.5
C3—C2—C1	121.00 (17)	H18B—C18—H18C	109.5
C3—C2—H2A	119.5	C15—C18—H18D	109.5
C1—C2—H2A	119.5	H18A—C18—H18D	141.1
C2—C3—C4	121.97 (18)	H18B—C18—H18D	56.3
C2—C3—H3A	119.0	H18C—C18—H18D	56.3
C4—C3—H3A	119.0	C15—C18—H18E	109.5
C3—C4—C5	116.52 (17)	H18A—C18—H18E	56.3
C3—C4—C7	119.74 (17)	H18B—C18—H18E	141.1
C5—C4—C7	123.73 (17)	H18C—C18—H18E	56.3
C6—C5—C4	122.11 (18)	H18D—C18—H18E	109.5
C6—C5—H5A	118.9	C15—C18—H18F	109.5
C4—C5—H5A	118.9	H18A—C18—H18F	56.3
C5—C6—C1	121.11 (18)	H18B—C18—H18F	56.3
C5—C6—H6A	119.4	H18C—C18—H18F	141.1
C1—C6—H6A	119.4	H18D—C18—H18F	109.5
C8—C7—C4	129.64 (18)	H18E—C18—H18F	109.5
C8—C7—C9	113.38 (17)	C20—C19—C24	119.55 (17)
C4—C7—C9	116.98 (17)	C20—C19—N1	119.58 (16)
C7—C8—C11	125.56 (19)	C24—C19—N1	120.84 (16)
C7—C8—C10	120.84 (19)	C21—C20—C19	119.90 (17)
C11—C8—C10	113.58 (19)	C21—C20—H20A	120.0
N2—C9—C7	178.5 (2)	C19—C20—H20A	120.0
N3—C10—C8	176.3 (2)	C20—C21—C22	121.67 (18)
N4—C11—C8	175.1 (2)	C20—C21—H21A	119.2
C13—C12—C17	120.05 (18)	C22—C21—H21A	119.2
C13—C12—N1	119.94 (17)	C23—C22—C21	117.49 (17)
C17—C12—N1	120.01 (18)	C23—C22—C25	120.97 (18)
C12—C13—C14	119.9 (2)	C21—C22—C25	121.54 (18)
C12—C13—H13A	120.1	C24—C23—C22	121.65 (18)
C14—C13—H13A	120.1	C24—C23—H23A	119.2
C15—C14—C13	121.5 (2)	C22—C23—H23A	119.2
C15—C14—H14A	119.2	C23—C24—C19	119.73 (18)
C13—C14—H14A	119.2	C23—C24—H24A	120.1
C14—C15—C16	117.84 (19)	C19—C24—H24A	120.1
C14—C15—C18	121.4 (2)	C22—C25—H25A	109.5
C16—C15—C18	120.7 (2)	C22—C25—H25B	109.5
C15—C16—C17	121.4 (2)	H25A—C25—H25B	109.5
C15—C16—H16A	119.3	C22—C25—H25C	109.5
C17—C16—H16A	119.3	H25A—C25—H25C	109.5
C12—C17—C16	119.3 (2)	H25B—C25—H25C	109.5
C12—C17—H17A	120.3		
			
C19—N1—C1—C6	7.9 (3)	C19—N1—C12—C17	−103.2 (2)
C12—N1—C1—C6	−172.42 (17)	C17—C12—C13—C14	−0.6 (3)
C19—N1—C1—C2	−173.19 (17)	N1—C12—C13—C14	178.40 (17)
C12—N1—C1—C2	6.5 (3)	C12—C13—C14—C15	0.6 (3)
N1—C1—C2—C3	−178.05 (17)	C13—C14—C15—C16	0.2 (3)
C6—C1—C2—C3	0.9 (3)	C13—C14—C15—C18	−179.2 (2)
C1—C2—C3—C4	−0.3 (3)	C14—C15—C16—C17	−1.0 (3)
C2—C3—C4—C5	−0.2 (3)	C18—C15—C16—C17	178.4 (2)
C2—C3—C4—C7	178.70 (17)	C13—C12—C17—C16	−0.1 (3)
C3—C4—C5—C6	0.1 (3)	N1—C12—C17—C16	−179.13 (17)
C7—C4—C5—C6	−178.76 (18)	C15—C16—C17—C12	0.9 (3)
C4—C5—C6—C1	0.5 (3)	C1—N1—C19—C20	−122.68 (19)
N1—C1—C6—C5	177.93 (18)	C12—N1—C19—C20	57.6 (2)
C2—C1—C6—C5	−1.0 (3)	C1—N1—C19—C24	59.2 (2)
C3—C4—C7—C8	−179.8 (2)	C12—N1—C19—C24	−120.46 (19)
C5—C4—C7—C8	−1.1 (3)	C24—C19—C20—C21	0.1 (3)
C3—C4—C7—C9	0.9 (3)	N1—C19—C20—C21	−178.05 (16)
C5—C4—C7—C9	179.71 (18)	C19—C20—C21—C22	−0.4 (3)
C4—C7—C8—C11	−0.9 (3)	C20—C21—C22—C23	0.1 (3)
C9—C7—C8—C11	178.3 (2)	C20—C21—C22—C25	179.62 (18)
C4—C7—C8—C10	177.54 (19)	C21—C22—C23—C24	0.5 (3)
C9—C7—C8—C10	−3.2 (3)	C25—C22—C23—C24	−178.97 (19)
C1—N1—C12—C13	−101.9 (2)	C22—C23—C24—C19	−0.9 (3)
C19—N1—C12—C13	77.7 (2)	C20—C19—C24—C23	0.6 (3)
C1—N1—C12—C17	77.1 (2)	N1—C19—C24—C23	178.66 (17)
